# Medical Malpractice Among Orthopedic Surgeons in Greece: An Analysis of Court Decisions

**DOI:** 10.7759/cureus.40243

**Published:** 2023-06-11

**Authors:** Nikolaos Baxevanos, Lambros Tzoumas, Marianna Korre, Konstantinos Tzoumas, Vasiliki Tzouma, Evangelia Samara, Georgios Papadopoulos

**Affiliations:** 1 9th Orthopedic Department, Metropolitan General Hospital, Athens, GRC; 2 Department of Anaesthesiology, University Hospital of Ioannina, Ioannina, GRC; 3 Department of Anesthesiology and Postoperative Intensive Care, Faculty of Medicine, School of Health Sciences, University of Ioannina, Ioannina, GRC

**Keywords:** manslaughter, court decisions, medical liability, medical malpractice, orthopedic surgeons

## Abstract

Introduction

Medical malpractice occurs when a physician, through incorrect medical action or omission, causes the patient to suffer physical harm or loss of life. Orthopedics is a high-risk medical specialty. Orthopedic surgery encompasses a wide range of procedures, including joint replacements, fracture repairs, and spinal surgeries. While orthopedic surgeons strive to provide optimal care to their patients, medical liability claims are a reality they must face. The aim of this study is to analyze the epidemiological data of judicial decisions and cases in Greece for the Specialty of Orthopedics.

Material and methods

Published court decisions involving medical liabilities of orthopedic surgeons and anesthesiologists, or only orthopedic surgeons were searched, in the period between 1985 and 2021. The judicial decisions were analyzed by an experienced anesthesiologist and an orthopedic surgeon based on medical knowledge and experience. Patients' age, gender, date of operation and the causes that led to the doctors’ persecution were also recorded.

Results

Seventy court decisions of the first, second, and third degree were found. These decisions related to 34 cases: seven convictions for manslaughter, 18 convictions for bodily harm, and nine acquittals - exempting compensation. They involved 11 men and 13 women. The claims mainly related to intraoperative and postoperative complications in 20 (83.3%) of the 24 cases. Complications in elective spinal and lower extremity surgeries represent 50% (n = 12) of cases, while postoperative complications account for 16.7% of cases (n = 4).

Conclusions

The present study concluded that an accumulation of experience in large orthopedic centers is needed to improve the performance of orthopedic surgeons during surgery and patient monitoring. Many legal cases are due to the mismatch between patient expectations and the limitations in medicine. Thorough preoperative control and better preoperative communication with the patient are needed, in order to improve the performance of orthopedic surgeons and prevent a significant part of the claims.

## Introduction

A surgical procedure can be dangerous not only for the patients, but it is also a risk for the surgeon in case of medical error, as well as for the anesthesiologist [1-3]. Medical malpractice occurs when a physician, through incorrect medical action or omission, causes the patient to suffer physical harm or loss of life. The problem of medical negligence is faced by all doctors in civilized countries. A study by Moreira et al. observed that more than 42% of all physicians have been prosecuted, with 57% of them being a surgical speciality [3].

Orthopedics is a high-risk medical specialty. Orthopedic surgeons play a critical role in diagnosing, treating, and managing musculoskeletal conditions. However, the nature of their profession exposes them to potential medical liability claims. According to Casali et al., 50% of orthopedic surgeons, who were the subject of a malpractice claim, had multiple claims against them until their retirement. In contrast, for the anesthesiologist, there was a 7% cumulative 10-year risk of being the subject of a malpractice claim and a 1% risk of being the subject of two or more claims [4].

The frequency of claims and the high compensations, especially in surgical specialties, have led in the USA to the crisis of "medical malpractice", a term that fully reflects the situation in which the country's Legal System has fallen, after the spectacular increase in medical malpractice claims [5-6]. And while the medical liability crisis was in full swing in the USA, other countries believed they were immune to medical malpractice liability problems. But they faced similar difficulties, albeit in delay [7].

To address the medical liability crisis, reforms have been made and analyses of closed claims for medical malpractice have been published [8]. Analyses of medical negligence cases have been used in the past to detect risk patterns and raise awareness of certain risks. If certain risk patterns are found, corrective or preventive action may be applied in future practice, minimizing the possibility of future medical errors.

Despite the enormous interest in medical malpractice, there is a significant lack of data on medical malpractice in Greece. The aim of this study is to evaluate the epidemiological data of judicial decisions and cases in Greece for the Specialty of Orthopedics, the causes that led to the poor outcome of patients and the amount of compensation paid, the criteria based on which they ended up in conviction or acquittal of orthopedic surgeons and the duration of the litigation until the final adjudication of the case.

## Materials and methods

Published court decisions of all levels and jurisdictions regarding medical liability at the criminal, civil, administrative, and disciplinary levels from 1980 to 2020 were searched in the legal information banks “Nomos”, “Sakkoulasonline.gr” and “Bank of the Athens Bar Association” and in legal magazines, such as “Nomiko Vima”, “Greek Justice”, “Criminal Chronicles”, “Criminal Justice”. The keywords “orthopedic”, “medical liability”, “negligence” and “medical error” were used. Decisions involving the medical liabilities of Orthopedic Surgeons and Anesthesiologists, or only Orthopedic Surgeons, were searched. Cases of bodily injury or manslaughter, in which the main responsibility was not derived by orthopedic surgeons, were excluded. Alleged medical errors or negligence for each decision were recorded along with the co-responsibility of the two doctors. Through these decisions, it was researched whether there is a fixed criterion for assigning responsibility and what is the basis for proving responsibility, when it is defined as a violation of a specific rule of medical science and art, and how jurisprudence defines the establishment of this rule and its sources.

The judicial decisions were analyzed by an experienced anesthesiologist and an orthopedic surgeon for the causes of death or bodily harm and the correctness of the court decision based on medical knowledge and experience. The lawyers undertaking this study contributed to the analysis of cases based on their law knowledge and experience. Patients' age, gender, date of operation and the causes that led to the doctors’ persecution were also recorded.

## Results

Seventy court decisions of the first, second, and third degree were found. These decisions related to 34 cases: seven convictions for manslaughter, 18 convictions for bodily harm, and nine acquittals - exempting compensation. Among the 24 conviction cases for manslaughter and negligent bodily harm, 10 were criminal, and 14 were civil. They involved 11 men and 13 women. The claims mainly related to intraoperative and postoperative complications in 20 (83.3%) of the 24 cases. There was wide variation in the causes that led to a medical malpractice claim. Complications in elective spinal and lower extremity surgeries represent 50% (n = 12) of cases, while postoperative complications account for 16.7% of cases (n = 4).

The criteria of the legal order on the basis of which the doctors involved were convicted of manslaughter and negligent bodily harm are depicted in Table 1.

**Table 1:** The criteria of the legal order on the basis of which the doctors involved were convicted of manslaughter and negligent bodily harm

Criteria	n	%
Poor handling or lack of attention during surgery leading to complications	12	50.0
Delayed treatment	4	16.7
Incomplete follow-up	2	8.3
Wrong or delayed diagnosis	2	8.3
Unnecessary surgery	1	4.2
Incomplete surgery	1	4.2
Insufficient preventive treatment	1	4.2
Failure to take a preoperative history	1	4.2
TOTAL	24	100

Table 2 shows the causes of manslaughter by orthopedic surgeons in criminal and civil cases. The average duration until the final adjudication of the case was 8.4 ± 2.41 years.

**Table 2:** Manslaughter due to negligence by orthopedic surgeons in criminal and civil cases

Medical complication	n
Delayed treatment of gastric bleeding due to gastric ulcer after intervertebral disc disease	1
Incorrect administration of local anesthetic with long-acting cortisone in an internal jugular vein because of musculoskeletal pain leading to heart attack	1
Vascular damage during cement placement	1
Postoperative myocardial infarction. Incorrect diagnosis of allergic shock (by telephone)	1
Meningitis following spinal fusion	1
Pulmonary embolism after anterior cruciate ligament surgery. Negligence of incorrect thromboprophylaxis	1
TOTAL	6

Table 3 shows cases of bodily harm due to negligence by orthopedic surgeons in criminal and civil cases. The average compensation was 123,767 ± 152,853 € with a range of 10.000 - 450.000 €.

**Table 3:** Bodily harm due to negligence by orthopedic surgeons in criminal and civil cases MTP joint: Metatarsophalangeal joint. Amounts in parentheses indicate the monetary damages awarded by the court.

Medical complication	n
Paraplegia caused by cauda equina syndrome after spinal decompression	1
Postoperative meningitis after spinal fusion	1
Spinal cord injury after spinal fusion	2
Popliteal artery injury in knee surgery	1
Tendon rupture leading to osteoarthritis of the 1^st^ MTP joint, after percutaneous osteotomy	1
Spinal cord injury and spinal root injury by spinal fusion screws, without proper indication of spinal fusion	1
Failure of removal of a benign tumor during spinal fusion	1
Failure of transfer of a paraplegic patient to a tertiary orthopedic center after a motor vehicle accident (300.000€)	1
Cauda equina syndrome after spinal decompression (230.000€)	1
Cauda equina syndrome after low back pain (130.000€)	1
Poor leg lengthening after hip arthroplasty (50.000€)	1
Unnecessary limb amputation in desmoid tumor removal surgery (450.000€)	1
Nerve injury after shoulder hemiarthroplasty (26.705€)	1
Nerve injury after removal of tibial hardware (40.000€)	1
Incorrect treatment of osteomyelitis (10.000€)	1
Testicular atrophy after hip adductors surgery (60.000€)	1
Peroneal nerve palsy after knee osteotomy (57.147€)	1
TOTAL	18

Orthopedic surgeons were acquitted in nine cases: 1. In a case of death from undiagnosed lung cancer after hip surgery; 2. In a case of death from undiagnosed posterior peritoneum bleeding after percutaneous kyphoplasty. The court found no medical error, as due to the nature and type of kyphoplasty as a closed procedure, the surgeon did not have the objective possibility to diagnose the bleeding in the posterior peritoneum, either macroscopically through the small skin holes of 1 cm, or fluoroscopically. Moreover, the patient's manifested hemodynamic instability, as a symptom, is compatible with causes other than bleeding, such as her allergic reaction to injection material (bone cement). Due to the aforementioned reasons, the surgeon could not take the medically indicated measures to diagnose and treat the bleeding; 3. In a case of wrist fracture malreduction; 4. In a case of death due to pulmonary embolism after femoral fracture fixation following a motor vehicle accident; 5. In a case of death due to allergic shock after intramuscular injection of diclofenac and thiocolchicoside and paraspinal injection of betamethasone and xylocaine. The orthopedic surgeon was accused of performing spinal anesthesia in a private office and of delaying transfer to a hospital. The death was attributed to an allergic reaction which could not be predicted as the patient had no history of allergy; 6. In a case of death in the Intensive Care Unit of a polytrauma patient, a victim of a terror attack; 7. In a case of death due to brain necrosis after a heart attack due to myocarditis, during flexor carpi ulnaris surgery; 8. In a case of deep venous thrombosis after knee arthroscopy. The patient had a history of thrombophilia, which hid did not know. The orthopedic surgeon diagnosed deep venous thrombosis and gave proper medical instructions; 9. In the case of death after acute kidney failure in scoliosis surgery.

## Discussion

In the present study, the incidence of manslaughter and bodily harm cases over a 35-year period is significantly low compared to the literature [4,9-12]. Compared to other surgical specialties in Greece, the medical negligence of orthopedic surgeons was approximately similar to that of anesthesiologists and significantly lower than that of gynecologists-obstetricians [13-14]. In the Traina F study, between the years 1996 and 2000, the orthopedic speciality was responsible for the largest number of cases submitted regarding alleged malpractice, followed by obstetrics/gynecology [15].

Our small number of cases compared to the literature may be explained by the fact of different legal systems existing in other countries, which may contribute to this discrepancy. Difficulties in the evidentiary difficulties of medical liability, different mentalities between people, and different patient-doctor relationships may play an important role. The fact is, as in other countries, there are many patients who have suffered medical malpractice but have not filed a claim. In the United States, it is estimated that only 1-3% of patients file a malpractice claim [16].

In 50% of cases of manslaughter and negligent bodily harm, there was insufficient performance or incorrect surgical technique or lack of care in elective operations of the spine and lower extremities (18%). The same incidence of medical negligence in spinal operations is presented by neurosurgeons in Greece. In the research of Samara et al., the incidence of medical negligence of neurosurgeons in spine operations was 75%. Among them, 46.67% concerned the lumbar and thoracic spine and 53.33% the cervical spine [17].

The results of the present study are in agreement with the published literature. In a study investigating the medical liability of Spanish neurosurgeons, more than 62% of the surgeries involved the spine, and about 29% affected the cranial region. Permanent postoperative consequences were reported in 40% of cases and death was reported in 22%. Inadequate documentation or consent was found in 17% of the lawsuits. One-fifth of the cases concerned criminal courts and four-fifths concerned civil courts [18]. In another similar study investigating the medical liability of neurosurgeons in the United States, the majority of these cases involved spinal surgeries. Manslaughter was observed in about 23% of patients. The main medical factor leading to a claim was insufficient neurosurgeon performance (42%), preoperative intervertebral disc disorder (21%), and spinal cord surgery (21%) [19]. In the study by Tarantino et al., including a total of 243 orthopedic claims, 149 (61%) were found to arise from elective surgery. Spine surgery and total hip arthroplasty were the most common surgical procedures. Insufficient informed consent was reported in 5.3% of cases [20].

A study investigating the medical liability of orthopedic surgeons in Britain concluded that the most usual causes of claims in orthopedics are post-operative complications, wrong, delayed or failed diagnosis, insufficient consent and surgery at the wrong site. Spinal surgery and lower extremity surgery have the most claims during elective surgery, while upper extremity surgery has the most claims during trauma surgery [11].

In our study, there were two (8.3%) claims regarding infections after spinal fusion. The problem of infections is particularly present when a foreign body is implanted and the incidence of infections can be as high as 14% of all claims [4]. In our investigation, there was a claim for orthopedic negligence in one case of paraplegia after a traffic accident, as the patient was not referred to an equipped orthopedic center. Claims in traumatology are probably attributed to the more superficial preoperative approach. One of the possible psychological causes of malpractice claims following trauma surgery is the absence of preoperative symptoms of illness or disability. Therefore, intraoperative surgical complications and failure of treatment are the most common starting points for a malpractice claim.

A recent study by Vasdeki et al. analyzed claims related to hand and wrist trauma in the Greek population for a 20-year period. Six cases were identified, including two convictions for bodily harm, with a mean compensation of 2000 €. However, we chose not to include these cases in our survey, as more medical specialities were involved, such as general surgeons and plastic surgeons [21].

A recent meta-analysis identified 13 studies investigating malpractice lawsuits in orthopedic surgery. More than 23000 claims related to orthopedic surgery were analyzed. Elective cases were more likely to result in lawsuits than trauma cases. Similarly to our study, intraoperative errors accounted for most malpractice claims. The most common reasons for litigation were suboptimal surgical performance, missed diagnosis and errors of informed consent [22].

In one case there was death from pulmonary embolism. Prevention of venous thrombosis and pulmonary embolism requires the administration of proper prophylaxis with appropriate medications and is the only way to reduce the risk to the patient and a malpractice claim. In the present study, there was an acquittal of orthopedic surgeons in nine complaints as no bad medical practice on their part was proven. In reality, however, they were punished since, as can be seen from the judicial data, most of the accused doctors were subjected to many years of legal suffering (the average duration of litigation is eight years), resulting in moral damage, the consumption of time, the degradation of their personality and significant financial burden. Medical liability claims can have far-reaching consequences for orthopedic surgeons. They not only result in financial losses due to legal fees and compensation but also have professional and reputational implications. Surgeons may experience increased premiums for malpractice insurance and damage to their professional standing. These factors can contribute to stress, burnout, and a decline in the overall quality of care.

A limitation of our research is the fact that there are unpublished court decisions, among others due to the long duration of a litigation. However, the analysis of the cases we presented is a useful tool in quality management in orthopedics and patient safety. It made it possible to review risk patterns to raise awareness of certain risks.

## Conclusions

The present study concluded that medical malpractice among orthopedic surgeons is not a rare phenomenon in Greece. Intraoperative errors and spinal surgeries were the most common factors involved in malpractice claims. Accumulation of experience in large orthopedic centers is needed to improve the performance of orthopedic surgeons during surgery and patient monitoring. Clear and open communication with patients is crucial. Thorough preoperative control and better preoperative communication with the patient are needed, in order to improve the performance of orthopedic surgeons and prevent a significant part of the claims. Surgeons should explain the risks, benefits, and alternatives of a procedure, ensuring patients understand the information provided. Obtaining informed consent is a vital step to reduce liability risks. At last, continuous medical education is crucial for the prevention of medical errors.
